# Comparison in Patients’ Adherence, Adverse Effects, and Clinical Outcomes Among Liver Transplant Recipients Using Immediate-Release Tacrolimus (Prograf^®^) Versus Extended-Release Tacrolimus (Advagraf^®^): First Report from Iran

**DOI:** 10.34172/aim.34896

**Published:** 2026-02-01

**Authors:** Mojtaba Shafiekhani, Mohamad Mahdi Mahmoudi, Hasan Rostami, Alireza Shamsaeefar, Sahar Sohrabi Nazari, Marziyeh Doostfatemeh, Fatemeh Alishavandi, Mohamad Sadegh Rajabian, Hamed Nikoupour

**Affiliations:** ^1^Department of Clinical Pharmacy, School of Pharmacy, Shiraz University of Medical Sciences, Shiraz, Iran; ^2^Thoracic and Vascular Surgery Research Center, Shiraz University of Medical Science, Shiraz, Iran; ^3^Shiraz Transplant Center, Abu-Ali Sina Hospital, Shiraz University of Medical Sciences, Shiraz, Iran; ^4^Department of Biostatistics, Shiraz University of Medical Sciences, Shiraz, Iran

**Keywords:** Adherence, Calcineurin inhibitors, Graft survival, Immunosuppression therapy, Liver transplantation

## Abstract

**Introduction:**

Medication adherence is a crucial factor in liver transplant patients’ improvement of quality of life and survival rates and reduction of allograft rejection. Tacrolimus plays a pivotal role in post-transplant immunosuppressive therapy, which is available both as twice-daily immediate-release tacrolimus (Prograf®) and once-daily extended-release tacrolimus (Advagraf®). This study compared adherence, adverse effects, and clinical outcomes between patients using once-daily extended-release tacrolimus (ERT; Advagraf®) and twice-daily immediate-release tacrolimus (IRT; Prograf®) in a real-world setting.

**Methods:**

This prospective observational study involved eligible adult liver transplant recipients at least six months post-transplant, prescribed either Prograf® or Advagraf® as part of their immunosuppressive regimen. The primary outcome was the change in adherence from baseline to six months. Patient adherence was assessed using the Basel Assessment of Adherence to Immunosuppressive Medication Scale (BAASIS) at baseline and 6 months. The dose-to-concentration ratio of tacrolimus, potential side effects, and intra-patient variability were assessed at baseline and at one-month and six-month follow-ups. Data were collected through face-to-face interviews, medical records review, pharmacy database exploration, and phone surveys.

**Results:**

Baseline adherence was similar between the groups (Prograf® 48.5% vs. Advagraf® 41.2%, *P*=0.39). At six months, the ERT (Advagraf®) group was associated with higher adherence rates in adjusted analysis (Adjusted Odds Ratio [AOR]: 2.5, 95% CI: 1.1-5.8). Factors significantly associated with higher adherence included older age (AOR: 1.05 per year, 95% CI: 1.01-1.09) and shorter time since transplant (AOR: 0.98 per month, 95% CI: 0.97-0.99). The ERT group also reported a lower incidence of hand tremors (15% vs. 32%, *P*=0.015). Trough levels, dose/concentration ratios, and renal function were comparable.

**Conclusion:**

In this observational study, the once-daily Advagraf® formulation was associated with significantly better medication adherence and a lower reported incidence of tremors compared to twice-daily Prograf®. These findings should be interpreted in the context of the study’s non-randomized design and potential confounding.

## Introduction

 Long-term immunosuppressive treatment after solid organ transplantation like liver transplantation (LT) is ideally maintained by treatments with stable pharmacokinetic profiles to improve quality of life and survival, and to reduce allograft rejection and graft loss.^1^ many solid-organ-transplant recipients do not correctly take their immunosuppressant therapy as prescribed, which can be subsequently a leading cause of graft rejection.^2-3^ Therefore, medication adherence is vital to achieve adequate immunosuppression. Compared to twice-daily dosing, once-daily treatment strategies can improve addressing non-adherence patients in routine clinical treatment.^4^ Hence, immunosuppressant compliance emerges as a crucial factor that may improve transplantation outcomes.

 Currently, the most common immunosuppressive medication practice protocol for solid-organ transplants generally includes a Calcineurin inhibitor (CNI), inhibiting T cells and impairing T-cell activation, proliferation, and differentiation.^5-7^ Tacrolimus is the most extensively preferred CNI for long-term maintenance after organ transplantation and is believed to have a more favorable impact on patient survival, graft loss, acute rejection, and steroid-resistant rejection.^5,8-9^ Tacrolimus is available both as twice-daily immediate-release tacrolimus (IRT; Prograf®) and once-daily extended-release tacrolimus (ERT; Advagraf®).^10-11^ Notably, ERT, with evidence of increased medication adherence, has been documented to significantly offer clinical advantages in solid organ transplant recipients and consequently improve patient outcomes.^4,10,12^

 The Abu-Ali Sina Organ Transplant Center, recognized as one of the largest transplant facilities globally, has transitioned to prescribing once-daily ERT for transplant recipients instead of the previously utilized twice-daily IRT since two years ago. This study aimed to compare the two formulations in a real-world clinical setting. The primary outcome was the change in medication adherence from baseline to six months, as measured by the BAASIS scale in accordance with safety, and clinical outcomes. This report represents the first study from Iran aimed at comparing adherence rates, adverse effects, and clinical outcomes reported following the transition from IRT to ERT in liver transplant recipients.

## Patients and Methods

###  Study Design 

 This prospective observational comparative study was conducted from November 2022 to November 2023 at the Abu-Ali Sina Organ Transplant Center in Shiraz, Iran, affiliated with Shiraz University of Medical Sciences. The study was approved by the ethics committee of Shiraz University of Medical Sciences with the IR.SUMS.REC.1401.398 code.

 The study involved liver transplant recipients deemed eligible based on the following inclusion criteria: 1) above 18 years of age, 2) receiving a liver transplant for the first time, 3) at least six months since their transplantation, and 4) prescribed tacrolimus by their physicians in either its extended-release (Advagraf®) or immediate-release (Prograf®) oral formulations as a component of their immunosuppressive regimen. Subjects were excluded from the study in case of receiving inconsistent daily doses of tacrolimus in a month prior to the commencement of the study, receiving pancreas or kidney transplantation concurrently, utilizing alternative brands of oral tacrolimus available in Iran’s pharmaceutical Markets besides Advagraf® and prograf®, or initiating Advagraf® immediately after surgery as a *de novo* approach. Additionally, we omitted participants with a confirmed history of hypersensitivity to tacrolimus or those who experienced acute transplant rejection less than four weeks before the study alongside patients with incomplete documentation or clinical/laboratory data. Patients who did not initiate or complete their medication regimen were not included in this study, as the focus is to compare the implementation of various formulations of oral tacrolimus.

 Tacrolimus dosing was performed according to center protocols, targeting trough levels of 5-10 ng/mL for patients beyond 6 months post-transplant. Dose adjustments were made by the treating physician or clinical pharmacist based on clinical assessment and trough levels. Also, trough levels were assessed immediately prior to the morning dose for both groups. For the Prograf® group, this was before the 12-hour dose; for the Advagraf® group, before the 24-hour dose. These through levels were measured by a chemiluminescence method using the ARCHITECT I system (Abbott, USA)

###  Patient Allocation and Study Groups

 Following a change in center policy two years prior to the study initiation, physicians were encouraged to prescribe ERT (Advagraf®) for all new transplant recipients and to consider converting stable patients from IRT (Prograf®). However, the final decision was at the physician’s discretion, influenced by drug availability, patient preference, and cost coverage. The Prograf® group thus consisted of patients who either underwent transplantation before this policy shift or for whom Prograf® was specifically continued. The Advagraf® group included patients converted from Prograf® and those initiated *de novo* on Advagraf® after the policy change. This non-random allocation introduces the potential for selection bias, as patients prescribed one formulation may differ systematically from those prescribed the other. To address this, we recorded the start date of the current formulation and used statistical methods to adjust for baseline differences.

 This study was reported in accordance with the Strengthening the Reporting of Observational Studies in Epidemiology (STROBE) guidelines.

###  Data Collection

 We gathered our participants’ data through face-to-face interviews, studied obtainable medical records at the clinic, explored archives in the Abu-Ali Sina Hospital pharmacy database, and conducted phone surveys with patients using pre-designed questionnaires. In conclusion, the following information was obtained from all subjects: demographical characteristics (age, gender), socioeconomic and cultural factors (marital status, place of residence, lifestyle habits, residential settings, educational backgrounds, employment status, drug addictions, alcohol consumption, smoking habits, and history of mental illnesses), clinical and laboratory data (cause of liver failure, type of donor, duration of transplant, serum tacrolimus levels (FK level), liver enzymes levels, estimated GFR (CKD-EPI-2021)), the employed immunosuppressive regimen and other administered medications, degree of motivation, compliance with the prescribed regimen and usage of reminder tools.

###  Evaluation of Patients’ Adherence to Tacrolimus Regimen

 Patients were assessed for their adherence to the tacrolimus regimen at the baseline and then again 6 months after using their medications over face-to-face or phone interviews via the Basel Assessment of Adherence to Immunosuppressive Medication Scale (BAASIS). BAASIS is a self-report questionnaire designed by the Lauven Basel adherence research group that assesses three main phases of medication adherence: initiation, implementation, and persistence.^13^ The scale assesses the implementation of immunosuppressives through four aspects of medication taking: dose taking, drug holiday, timing adherence, and dose reduction (Table 1). Responding “Yes” to even a single inquiry regarding the past four weeks was classified as non-adherence. Reminder tools in any form, such as timed pillboxes, calendars, and phone applications, were documented alongside patients’ possible reasons for not adhering to their prescribed regimen.

**Table 1:** Basel Assessment of Adherence to Immunosuppressive Medication Scale – Implementation Phase Questionnaire.

**Evaluated component**	**Questions**	**Acceptable answers**
Dose taking	Do you recall not taking your tacrolimus capsules at any time during the past four weeks?	Yes / No
Drug holiday	Have you skipped several consecutive doses of tacrolimus during the past four weeks?	Yes / No
Timing adherence	Do you recall taking your tacrolimus capsules more than two hours from the prescribed dosing time during the past four weeks?	Yes / No
Dose reduction	Have you reduced the prescribed amount of your tacrolimus in the past four weeks?	Yes / No

###  Assessment of Adverse Effects

 Adverse events were assessed through direct, structured questioning at each monthly visit using a predefined checklist of known tacrolimus side effects, its causality and grading. Events were documented as present or absent based on patient self-report and review of systems.

###  Measurements and Calculations 

####  Calculation of Tacrolimus Dose to Concentration Ratio

 We measured and calculated the tacrolimus dose-to-concentration ratio at the baseline and, subsequently, one month and six months after initiation in both groups. The tacrolimus level was adjusted by physicians, if necessary.

####  Calculation of Tacrolimus Intra-patient Variability (IPV)

 Intra-patient variability is the fluctuations in tacrolimus concentration observed within the same patient over a specified period despite constant dosage. In this study, IPV was calculated and compared between the two groups at the baseline and one month and 6 months after using the coefficient of variation (CV).^14^ Intra-patient variability (IPV) was calculated as the coefficient of variation (CV = [Standard Deviation / Mean] × 100%) for all available trough levels during the study period.

###  Statistical Analysis

 Continuous variables were summarized as mean ± standard deviation or median with interquartile range according to their distribution, and categorical variables as frequencies and percentages. Baseline comparisons between the two treatment groups (Prograf vs. Advagraf) were performed using independent samples t-test or Mann–Whitney U test for continuous variables, and chi-square test or Fisher’s exact test for categorical variables, as appropriate.

 Given the repeated-measures design and within-patient correlation of observations, all longitudinal analyses were conducted using Generalized Estimating Equations (GEE). This approach was applied uniformly to both binary and continuous outcomes to obtain population-averaged effects.

 Binary outcomes (including overall medication adherence at baseline and 6 months, as well as all BAASIS-derived adherence components [dose omissions, repeated omissions, timing deviations, and dose reductions]) were modeled using GEE with a binomial distribution and logit link. Each model incorporated fixed effects for treatment group, time, and the group-by-time interaction. Prespecified covariates (age, sex, time since transplantation, number of prescribed medications, marital status, and education level) were included for adjustment. Results were reported as odds ratios with 95% confidence intervals and Wald *P*-values. For interpretation of significant interactions, marginal predicted probabilities were computed and presented graphically.

 Continuous biochemical outcomes (serum creatinine, estimated glomerular filtration rate (GFR), AST, ALT, and ALP) measured at baseline, 1-month, and 6-month visits, were analyzed using GEE with a Gaussian family and identity link. Models included treatment group, time, their interaction, and the same covariates applied in the binary analyses. Estimated marginal means were used to derive fitted time-trajectories, which were visualized to illustrate temporal patterns and potential differential effects between formulations.

 For all outcomes, interaction terms were further examined using contrast analyses to characterize pairwise group differences across time points. These contrasts, together with interaction plots, provided complementary and clinically interpretable summaries of longitudinal effects.

 All analyses were conducted using R (version 4.5.0), employing the geepack package for model estimation and ggplot2 for graphical visualization. A two-sided *P*-value < 0.05 was considered statistically significant.

## Results

 In our study, 150 patients were screened, and 16 patients were excluded due to re-transplantation or drug change during 6 months after the first operation. Among the remaining 134 patients, 66 were prescribed twice daily Prograf®, and 68 received once daily Advagraf®. The demographic, clinical, and socio-economic-cultural characteristics of all patients are shown in Table 2. The majority of included patients were male (54.8%), with ages ranging from 24 to 85 years (mean ± standard deviation; 50.20 ± 13.08). The most common etiologies of liver cirrhosis in our study were primary sclerosing cholangitis (PSC) (23.7%), cryptogenic (15.5%), non-alcoholic steatohepatitis (NASH) (14.8%), and autoimmune hepatitis (AIH) (14.81%).

**Table 2:** Demographic, Clinical, and Socio-economic-Cultural Characteristics of the Liver Transplant Recipients Receiving Prograf® or Advagraf®.

**Variables**		**Total ** **(N=134)**	**Prograf®** **(N=66)**	**Advagraf®** **(N=68)**	***P*** **- value**
Gender; (%)	Male	74 (54.8)	41 (62.1)	33 (48.5)	0.11
	Female	60 (45.2)	25 (37.9)	35 (51.5)	
Age (years); (mean ± SD)	50.20 ± 13.08 (24-85)	49.75 ± 14.34 (24-85)	50.64 ± 11.82 (26-73)	0.71
Etiology of liver failure; (%)	PSC	32 (23.7)	17 (25.8)	15 (22.11)	0.87
	Cryptogenic	21 (15.5)	7 (70.6)	14 (20.6)	
	NASH	20 (14.81)	8 (12.1)	12 (17.6)	
	AIH	20 (14.81)	9 (13.2)	11 (16.2)	
	HBV/HCV	18 (13.33)	9 (13.2)	9 (13.6)	
	BCS	9 (6.66)	7 (10.6)	2 (2.9)	
	ALF	3 (2.22)	1 (1.5)	2 (2.9)	
	HCC	1 (0.71)	1 (1.5)	0	
	Alcoholic	5 (3.70)	4 (6.1)	1 (1.5)	
	Wilson	3 (2.22)	2 (3)	1 (1.5)	
	N.A	2(1.49)	0(0)	2 (2.9)	
Mean duration since transplant (months); mean ± SD		85.61 ± 50.21	90 ± 50.84	81.22 ± 49.58	0.39
Number of prescribed medications;		6.32 ± 2.23	6.12 ± 2.27	6.53 ± 2.18	0.18
Education; (%)	Illiterate	16 (11.85)	5 (7.6)	11 (16.2)	0.21
	School degree	90 (66.66)	46 (69.7)	44 (64.7)	
	Academic degree	28 (20.74)	15 (22.7)	13 (19.11)	
Drug Addiction; (%)	Never	130 (96.29)	64 (97)	66 (97.1)	0.97
	Previously	4 (3.61)	2 (3)	2 (2.9)	
	Currently	0	0	0	
Alcohol Consumption; (%)	Never	123 (91.11)	59 (89.4)	64 (94.1)	0.32
	Previously	11 (8.89)	7 (10.6)	4 (5.9)	
	Currently	0	0	0	

PSC: Primary Sclerosing Cholangitis, NASH: Non-Alcoholic Steatohepatitis, AIH: Auto Immune Hepatitis, HBV/HCV: Hepatitis B virus/Hepatitis C virus, BDS: Budd-Chiari syndrome, ALF: Acute liver failure, HCC: hepatocellular carcinoma, N.A: not available.

 Based on BAASIS criteria, baseline adherence in the Prograf® and Advagraf® groups were 32 (48.48%) and 28 (41.17%), respectively, with no significant difference (*P* = 0.39). The majority of patients (96%) had adequate motivation to resume treatment. Moreover, 75.46% of patients had their spouses or children assisting them in taking their medication on time. Approximately 18% of the study population reported using reminders, with alarm clocks used the most commonly. The most common reasons for non-adherence with Prograf® and Advagraf® were forgetfulness and medication shortages/unavailability, respectively.

 As presented in Table 3, in the adjusted model, neither treatment group nor time alone was significantly associated with adherence. At baseline, adherence did not differ significantly between patients receiving Advagraf and those receiving Prograf (OR = 0.59, 95% CI 0.28–1.26; *P* = 0.17). Similarly, no overall change in adherence from baseline to 6 months was observed in the reference group (Prograf) (OR = 1.07, 95% CI 0.59–1.95; *P* = 0.82).

**Table 3:** Multivariate GEE Model of Independent Qualitative and Quantitative Factors of Adherence.

**Variable**	**B**	**SE**	***P*** **-value**	**Odds Ratio**	**95% CI**
Group(Advagraf)	-0.520	0.381	0.173	0.595	(0.282, 1.256)
Time(6month)	0.070	0.305	0.818	1.073	(0.589, 1.952)
Age(year)	0.000	0.014	0.99	1.000	(0.974, 1.027)
Sex(Female)	-0.162	0.297	0.585	0.850	(0.475, 1.522)
Time since transplantation (months)	-0.015	0.003	< 0.001	0.986	(0.979, 0.992)
Number of prescribed medications	-0.008	0.071	0.911	0.992	(0.864, 1.14)
Marital status(Married)	-0.554	0.432	0.199	0.574	(0.246, 1.339)
Education(School)	-0.786	0.557	0.158	0.456	(0.153, 1.358)
Education(Academic)	-0.856	0.655	0.191	0.425	(0.118, 1.533)
Group*Time	1.169	0.449	0.009	3.218	(1.335, 7.758)
contrast		estimate	se	p	CI
Group: Advagraf vs Prograf at Baseline		-0.520	0.381	0.173	(-1.267, 0.228)
Group: Advagraf vs Prograf at 6 months		0.649	0.384	0.091	(-0.103, 1.401)
Time: 6m vs Baseline in Prograf		0.070	0.305	0.818	(-0.529, 0.669)
Time: 6m vs Baseline in Advagraf		1.239	0.330	< 0.001	(0.593, 1.885)

 However, a statistically significant interaction between treatment group and time was detected (OR = 3.22, 95% CI 1.34–7.76; *P* = 0.009), indicating that changes in adherence over time differed between the two tacrolimus formulations. Contrast analyses were conducted to clarify this interaction. These analyses showed no significant difference between groups at baseline, and no significant temporal change in adherence within the Prograf group. In contrast, patients receiving Advagraf demonstrated a marked and statistically significant increase in adherence from baseline to 6 months (*P* < 0.001).

**Figure 1:**
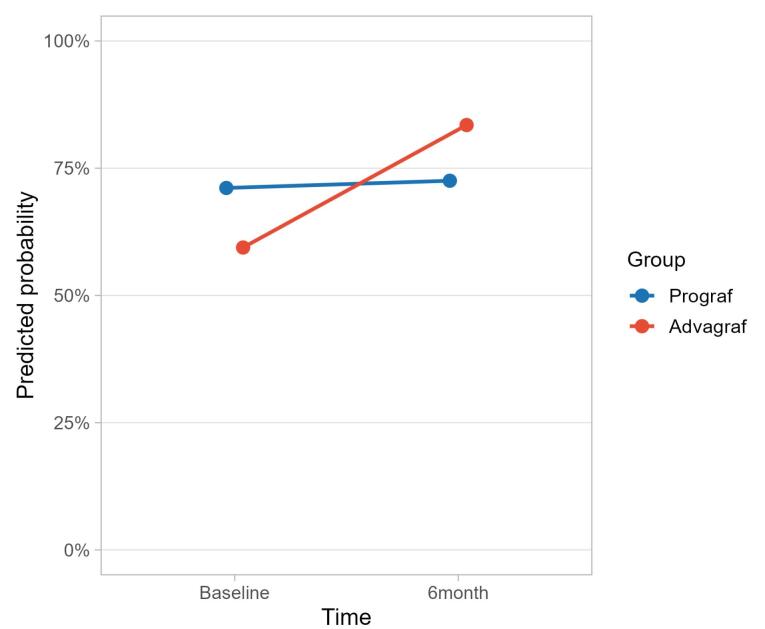
Interaction Plot of Medication Adherence over the Time by Treatment Group

 These findings are illustrated in the interaction plot (Figure 1), which shows relatively stable adherence probabilities over time in the Prograf group, compared with a pronounced improvement in adherence among patients treated with Advagraf. Together, these results indicate that while overall adherence levels were comparable at baseline, the once-daily Advagraf formulation was associated with a significant improvement in adherence over time, whereas adherence remained unchanged among patients receiving Prograf.

 Data regarding association between continuous biochemical outcomes in the follow up period are provided in Supplementary Tables 1A-D. In terms of dimensions from the BAASIS scale in both the Prograf® and Advagraf® groups at baseline and after six months, forgetting consecutive drug doses, more than two hours delay in drug consumption, and correct drug consumption factors were statistically significant with a *P*-value of 0.006, < 0.001, and < 0.001 respectively (Table 4) (Supplementary Tables 2A-D).

**Table 4:** Adherence Scale of Liver Transplant Patients under Treatment with Prograf® and Advagraf® at Baseline and 6 Months Later.

**BAASIS scale criteria**		**At baseline**		**After six months**		***P*** ** value**
		**Yes**	**No**	**Yes**	**No**	
Drug dose forgetting	Prograf®	18	48	21	45	0.43
	Advagraf®	23	45	17	51	0.22
Forgetting consecutive drug doses	Prograf®	7	59	3	63	0.15
	Advagraf®	10	58	1	67	0.006
> 2 hours delay in drug consumption	Prograf®	25	41	26	40	0.79
	Advagraf®	32	36	12	56	< 0.001
Correct drug consumption	Prograf®	14	52	8	51	0.79
	Advagraf®	18	50	4	64	< 0.001

 During the six-month observation period, no mortality was reported in either the Advagraf® or the Prograf® group. There were no reported acute rejection episodes in the Advagraf® compared to the Prograf® group. Baseline serum aspartate transaminase (AST) and alanine transaminase (ALT) were compared between the Prograf® and Advagraf® groups in both baseline and after six months, which did not change during the observation period. However, baseline serum alkaline phosphatase (ALP) levels were compared in both groups, and the Prograf® group experienced a reduction in serum levels in month 6. In comparison to baseline values (month 0), the Advagraf® group experienced an increase in mean serum ALP levels in month 6. This difference was statistically significant (*P* value = 0.01). Through a period of six months, an increase in baseline serum creatinine level and estimated glomerular filtration rate (eGFR) was observed in both groups; however, the difference in serum creatinine level and eGFR between Prograf® and Advagraf® recipients after 6 months was not statistically significant (*P* value = 0.64, 0.16; respectively)

 Regarding intra-patient variability (IPV) over a six-month duration, the IPV in the Advagraf® group (34.9 ± 32.39) was lower than that in the Prograf® group (36.02 ± 43.52); however, this difference did not reach the level of statistical significance (*P* value = 0.71). Moreover, although the IPV value was lower in the adherent group (34.38 ± 34.49) than in the non-adherent group after six months (36.90 ± 41.97), this difference was also not statistically significant (*P* value = 0.79).

 In addition, the tacrolimus trough level, mean daily dose, and dose/concentration ratio were comparable between the Advagraf® and Prograf® groups at baseline, month one, and month six, and showed no statistically significant difference (Table 5). This demonstrates that using either once daily Advagraf® or twice daily Prograf® yields a similar target dose/concentration.

**Table 5:** Comparison of trough level, mean daily dose, and dose/concentration between Prograf® and Advagraf® recipients at the baseline, and after one and six months.

**Time**	**Tacrolimus formulation**	**Trough level (ng/dl; mean±SD)**	***P*** ** value**	**Mean daily dose** **(mg; mean±SD)**	***P*** ** value**	**Dose/concentration ratio**	***P*** ** value**
Baseline	Prograf®	6.24 ± 2.54	0.22	2.03 ± 1.06	0.38	3.07	0.11
	Advagraf®	5.61 ± 3.20		2.45 ± 0.95		2.28	
After one month	Prograf®	6.15 ± 2.80	0.20	2.18 ± 1.05	0.16	2.82	0.91
	Advagraf®	5.60 ± 2.80		2.42 ± 0.91		2.30	
After six months	Prograf®	5.90 ± 2.66	0.63	2.18 ± 1.05	0.18	2.70	0.31
	Advagraf®	5.65 ± 3.03		2.39 ± 0.94		2.36	

 In terms of adverse effects, Advagraf® showed a significantly lower incidence of hand tremors during the six-month observation (*P* value = 0.015) according to self-reports. Other neurotoxic side effects, such as headache or seizure, showed no significant differences between the two groups (*P* values = 0.06 and 0.43, respectively).

## Discussion

 In this prospective observational cohort, the use of once-daily extended-release tacrolimus (Advagraf®) was associated with a significant improvement in self-reported medication adherence over six months compared to the twice-daily formulation (Prograf®), after adjusting for key patient characteristics. Numerous studies have highlighted the role of post-transplant immunosuppressive therapy as a crucial factor concerning graft survival.^15-17^ Tacrolimus is often considered a pivotal component among immunosuppressive medications as it demonstrates significant efficacy in reducing transplant rejection while decreasing the required corticosteroid dosage in transplant recipients.^17-18^ Considering the importance of an optimal immunosuppressive regimen, this study represents the first comparative analysis of patients receiving extended versus immediate formulations of oral tacrolimus in Iran. Our investigation demonstrated that utilizing Advagraf® can promisingly affect adherence rates while maintaining a comparable dose/concentration ratio.

 Several studies have explored the feasibility of converting from IRT (Prograf®) to its extended-release formulations in liver transplant recipients. Cassuto *et al*. observed improved compliance in 20% of kidney and liver recipients after transitioning from Prograf® to Advagraf^®^.^19^ Comparable findings were reported in studies conducted in Sweden and the United States where, respectively, 19% and 37.4% of participants reported better adherence after the conversion to ERT (Advagraf®).^20-21^ While we did not observe any noteworthy changes among patients utilizing Prograf, the adherence rates were significantly enhanced in the Advagraf® group (increasing from 41.17% to 67.64% throughout the 6-month study period, *P* < 0.05). These consistent findings across different healthcare settings suggest the reliable advantage of ERT (Advagraf®) in terms of adherence improvement.

 We observed that individuals receiving Advagraf® were significantly more adherent to the time of their medication. Additionally, subjects within this group were less likely to miss multiple consecutive doses of medication. These findings are in coherence with previous reports introducing forgetfulness in taking medicine and delays in administration as two significant factors contributing to non-adherence.^21-24^ Patients tend to modify the timing of their medication to accommodate their work schedules and meal plans or even for reasons such as vacations or stress.^25^ Additionally, many transplant patients suffer from various comorbidities, which necessitate the use of multiple medications throughout the day. This may add to the complexity of medication management and render adherence more challenging. The transition from twice-daily Prograf® to once-daily Advagraf® can aid patients with the burden of time management, potentially explaining the improved adherence rates. This suggests that reducing medication intake frequency will give patients more flexibility in their daily routine, positively impacting their cooperation with the prescribed medications.

 In addition to medication formulation, younger age and time since transplant emerged as significant factors influencing non-adherence. Various studies have explored factors influencing patients’ adherence, and there is a consensus in the current literature concerning the susceptibility of younger adults and recipients who have had their graft for a long time to non-adherence.^22,26,27^ This is interestingly contrary to common expectations as it is widely assumed that older patients are more prone to forgetting their medications or that as time passes, patients are likely to incorporate medications into their daily routines. A comprehensive 10-year follow-up study by Toti *et al.* demonstrated that while non-adherence significantly reduced after six months of converting to prolonged-release medication, there was a slight increase in subsequent years, especially regarding timing adherence and drug holidays.^24^

 Possible psychological factors may be attributed to this pattern. Patients often experience a sense of renewed well-being following a transplant, which can lead to the misconception that they are entirely cured. This belief may result in diminished commitment to following their physicians’ instructions.^25^ Moreover, the prospect of dependence on lifelong therapy often causes a sense of overwhelming feelings, especially in younger patients.^25^ Understanding these psychological barriers is crucial in developing effective treatment strategies.

 Our study compared IPV and the dose-to-concentration ratio between the two groups. However, it is essential to note that IPV is not a reliable measurement for assessing patients’ adherence, given that it could be easily influenced by a range of factors unrelated to adherence, including interactions between food and medications, genetic factors such as CYP 3A4 and 3A5 polymorphism, and gastrointestinal issues like diarrhea^28^. Nonetheless, it has been demonstrated that tacrolimus has lower intra-patient variability in highly adherent patients.^29^ Additionally, studies showed a correlation between high intra-patient variability, particularly above 35%, and unfavorable outcomes^30^ Coinciding with these findings, no significant correlation between IPV and patients’ adherence was observed in our study. Furthermore, while the difference may be insignificant, patients receiving Advagraf® exhibited lower IPV. Conversely, in their prospective 10-year study, Toti *et al*. demonstrated a significant decrease in IPV after converting patients from immediate to prolonged-release tacrolimus.^24^ While we are uncertain about the exact cause behind these discrepant findings, the factor of time may have a role in this context as another study by Bunthof *et al.* also demonstrated insignificant changes in IPV after conversion to prolonged formulations of tacrolimus in a 6-month follow-up study.^31^

 Our study found a significant increase in alkaline phosphatase levels among subjects receiving Advagraf® (from 140.08 ± 210.7 to 213 ± 260.19, *P*-value ≤ 0.05). The existing data on the hepatotoxic aspect of tacrolimus is limited, and the exact underlying mechanism is unknown, yet the observed pattern appears to be predominantly cholestatic.^32-33^ Lv *et al.* reported young age, low body weight, and abnormal alkaline phosphatase levels at the baseline as risk factors for tacrolimus-induced hepatotoxicity.^32^ The increase in alkaline phosphatase levels in our study did not reach hepatotoxic levels. However, we observed that in coherence with Lv’s work, there were slightly higher baseline alkaline phosphatase levels among the Advagraf® group.

 There is a conflict in the literature regarding the extent to which a higher dosage of tacrolimus contributes to the mechanisms underlying hepatotoxicity.^33-34^ Our study observed no significant difference in the dose-to-concentration ratio between the two formulations of tacrolimus. Thus, it is possible that other factors contribute here. Given the rarity of this phenomenon, further investigation is essential to elucidate the specific underlying mechanisms involved.

 A critical consideration in interpreting our results is that the majority of self-report tools for assessing medication adherence are designed based on the healthcare frameworks and infrastructure found in high-income countries.^35^ While it has been established that financial constraints may result in medication trade-offs that impede adherence, it is crucial to recognize the role of the barriers the patients may face to access the medication even when they manage to collect financial resources.^36^ In our study, the main reason for non-adherence in the Advagraf® group was the difficulty patients encountered in acquiring the medicine. Due to the challenges associated with accessing the treatment, patients reported frequently missing doses or having to go through drug holidays. This may suggest a potentially higher adherence rate within this group in the context of stability in the pharmaceutical market. These findings highlight the need for policy interventions to improve medication accessibility and distribution networks. Additionally, physicians should take this factor into consideration when evaluating the optimal pharmacotherapy for their patients.

 Overall, according to previous studies, converting from Prograf® to Advagraf® is demonstrated to be safe and efficient.^24^ Advagraf® has been shown to boost the quality of life in liver transplant recipients by reducing the burden of a strict dosing schedule [19, 24]. Studies also suggest ERT (Advagraf®) provides greater bioavailability, lower peak levels, and diminished overall blood exposure to tacrolimus. These factors may contribute to the reduced incidence of tremors experienced by patients and improvements in kidney function while efficiently protecting patients against transplant rejection. ^24,37-38^

 Our findings must be interpreted in the context of several important limitations. First, the non-randomized design is susceptible to selection bias and unmeasured confounding. Although we used statistical methods to adjust for observed baseline differences, unmeasured factors (e.g. underlying patient motivation) may influence both the formulation prescribed and adherence outcomes. Second, the per-protocol inclusion criteria, which excluded patients who changed drugs or were non-adherent at the outset, may limit the generalizability of our results to a more unstable population. Third, adherence was measured by self-report, which is subject to social desirability bias and may overestimate true adherence. The lack of objective adherence measures (e.g. electronic monitoring) is a notable weakness. Furthermore, our adverse event assessment relied on patient self-report without independent adjudication, which risks under-ascertainment and limits the generalizability of our safety findings. Finally, the single-center nature and relatively short follow-up period preclude conclusions about long-term graft outcomes.

## Conclusion

 In conclusion, the results of our study provide a better understanding of the possible differences between the two formulations of oral tacrolimus for the first time within the Iranian population. Our findings demonstrate that compared to IRT (Prograf®), a once-daily ERT(Advagraf®) improved adherence among liver transplant recipients, particularly regarding timing adherence and consecutive dose maintenance with same efficacy.

## Supplementary File


Table 1-A: Comparison of Alanine aminotransferase between tacrolimus formulation groups over six months using Generalized Estimating Equations (GEE) analysis
Table 1-B: Comparison of aspartate aminotransferase between tacrolimus formulation groups over six months using Generalized Estimating Equations (GEE) analysis
Table 1-C: Comparison of alkaline phosphatase between tacrolimus formulation groups over six months using Generalized Estimating Equations (GEE) analysis
Table 1-D: Comparison of estimated GFR between tacrolimus formulation groups over six months using Generalized Estimating Equations (GEE) analysis
Table 2-A: Comparison of BAASIS scale criteria (Drug dose forgetting) groups over six months using Generalized Estimating Equations (GEE) analysis
Table 2-B: Comparison of BAASIS scale criteria (Forgetting consecutive drug doses) groups over six months using Generalized Estimating Equations (GEE) analysis
Table 2-C: Comparison of BAASIS scale criteria (>2 hours delay in drug consumption) groups over six months using Generalized Estimating Equations (GEE) analysis
Table 2-D: Comparison of BAASIS scale criteria (Correct drug consumption) groups over six months using Generalized Estimating Equations (GEE) analysis
