# Supplementary material for: Comparison in Patients’ Adherence, Adverse Effects, and Clinical Outcomes Among Liver Transplant Recipients Using Immediate-Release Tacrolimus (Prograf®) Versus Extended-Release Tacrolimus (Advagraf®): First Report from Iran
Source: Arch Iran Med. 2026 Feb 1;29(2):77–85. doi: 10.34172/aim.34896 (PMC13477564; doi:10.34172/aim.34896)
Supplement: Supplementary file 1 [file aim-29-77-s001.pdf]

Arch Iran Med. February 2026;29(2):77-S1

doi:10.34172/aim.34896

http://journalaim.com

Supplementary Tables 1A-D. Association between continuous biochemical outcomes in the follow up period.

Supplementary Table 1A. Comparison of Alanine aminotransferase between tacrolimus formulation groups over six months using Generalized Estimating Equations (GEE) analysis.

Comparison	Estimate	SE	p	CI
Group: Advagraf vs. Prograf at Baseline	-2.780	3.59	0.438	(-9.811, 4.251)
Group: Advagraf vs. Prograf at 1 month	2.392	7.38	0.746	(-12.069, 16.852)
Group: Advagraf vs. Prograf at 6 months	-1.994	3.77	0.597	(-9.391, 5.403)
Time: 1m vs. Baseline in Prograf	2.398	2.76	0.385	(-3.01, 7.807)
Time: 6m vs. Baseline in Prograf	-1.174	3.45	0.734	(-7.943, 5.596)
Time: 1m vs. Baseline in Advagraf	7.570	6.20	0.222	(-4.585, 19.726)
Time: 6m vs. Baseline in Advagraf	-0.387	2.53	0.878	(-5.344, 4.569)

Supplementary Table 1B. Comparison of aspartate aminotransferase between tacrolimus formulation groups over six months using Generalized Estimating Equations (GEE) analysis.

Comparison	Estimate	SE	p	CI
Group: Advagraf vs. Prograf at Baseline	-1.013	2.27	0.655	(-5.462, 3.435)
Group: Advagraf vs. Prograf at 1 month	0.590	4.06	0.884	(-7.364, 8.545)
Group: Advagraf vs. Prograf at 6 months	-0.395	2.21	0.858	(-4.722, 3.932)
Time: 1m vs. Baseline in Prograf	1.839	1.86	0.322	(-1.805, 5.484)
Time: 6m vs. Baseline in Prograf	-0.521	1.50	0.729	(-3.468, 2.426)
Time: 1m vs. Baseline in Advagraf	3.443	3.85	0.371	(-4.107, 10.993)
Time: 6m vs. Baseline in Advagraf	0.097	1.59	0.951	(-3.01, 3.205)

Supplementary Table 1C. Comparison of alkaline phosphatase between tacrolimus formulation groups over six months using Generalized Estimating Equations (GEE) analysis.

Comparison	Estimate	SE	p	CI
Group: Advagraf vs. Prograf at Baseline	-8.43	21.38	0.693	(-50.34, 33.478)
Group: Advagraf vs. Prograf at 1 month	-12.92	23.65	0.585	(-59.282, 33.436)
Group: Advagraf vs. Prograf at 6 months	37.41	28.63	0.191	(-18.696, 93.519)
Time: 1m vs. Baseline in Prograf	12.47	10.29	0.225	(-7.694, 32.645)
Time: 6m vs. Baseline in Prograf	4.44	12.00	0.712	(-19.085, 27.958)
Time: 1m vs. Baseline in Advagraf	7.98	9.29	0.390	(-10.228, 26.195)
Time: 6m vs. Baseline in Advagraf	50.28	17.88	0.005	(15.226, 85.331)

Supplementary Table 1D. Comparison of estimated GFR between tacrolimus formulation groups over six months using Generalized Estimating Equations (GEE) analysis.

Comparison	Estimate	SE	p	CI
Group: Advagraf vs. Prograf at Baseline	-3.335	4.80	0.487	(-12.742, 6.071)
Group: Advagraf vs. Prograf at 1 month	1.870	4.22	0.658	(-6.396, 10.136)
Group: Advagraf vs. Prograf at 6 months	-3.692	4.86	0.447	(-13.218, 5.833)
Time: 1m vs. Baseline in Prograf	-5.296	3.35	0.114	(-11.861, 1.269)
Time: 6m vs. Baseline in Prograf	-0.865	3.70	0.815	(-8.11, 6.38)
Time: 1m vs. Baseline in Advagraf	-0.091	1.83	0.961	(-3.683, 3.501)
Time: 6m vs. Baseline in Advagraf	-1.222	2.39	0.609	(-5.9, 3.456)

Supplementary Tables 2A-D. Comparison of BAASIS scale criteria between the groups over six months using Generalized Estimating Equations analysis.

Supplementary Table 2A. Comparison of BAASIS scale criteria (Drug dose forgetting) between the groups over six months using Generalized Estimating Equations (GEE) analysis.

Comparison	Estimate	SE	p	CI
Group: Advagraf vs. Prograf at Baseline	0.586	0.646	0.364	(-0.68, 1.852)
Group: Advagraf vs. Prograf at 6 months	-1.058	1.206	0.380	(-3.421, 1.305)
Time: 6m vs. Baseline in Prograf	-0.994	0.714	0.164	(-2.393, 0.406)
Time: 6m vs. Baseline in Advagraf	-2.638	1.117	0.018	(-4.828, -0.448)

Supplementary Table 2B. Comparison of BAASIS scale criteria (Forgetting consecutive drug doses) between the groups over six months using Generalized Estimating Equations (GEE) analysis.

Comparison	Estimate	SE	p	CI
Group: Advagraf vs. Prograf at Baseline	0.823	0.366	0.025	(0.105, 1.541)
Group: Advagraf vs. Prograf at 6 months	-1.028	0.419	0.014	(-1.849, -0.207)
Time: 6m vs. Baseline in Prograf	0.070	0.272	0.797	(-0.463, 0.603)
Time: 6m vs. Baseline in Advagraf	-1.781	0.361	<0.001	(-2.489, -1.074)

Supplementary Table 2C. Comparison of BAASIS scale criteria (>2 hours delay in drug consumption) between the groups over six months using Generalized Estimating Equations (GEE) analysis.

Comparison	Estimate	SE	p	CI
Group: Advagraf vs. Prograf at Baseline	0.498	0.379	0.189	(-0.245, 1.241)
Group: Advagraf vs. Prograf at 6 months	-0.214	0.411	0.603	(-1.019, 0.592)
Time: 6m vs. Baseline in Prograf	0.241	0.307	0.433	(-0.361, 0.842)
Time: 6m vs. Baseline in Advagraf	-0.471	0.388	0.225	(-1.232, 0.29)

Supplementary Table 2D. Comparison of BAASIS scale criteria (Correct drug consumption) between the groups over six months using Generalized Estimating Equations (GEE) analysis.

Comparison	Estimate	SE	p	CI
Group: Advagraf vs. Prograf at Baseline	0.715	0.466	0.125	(-0.199, 1.628)
Group: Advagraf vs. Prograf at 6 months	-0.324	0.762	0.671	(-1.817, 1.17)
Time: 6m vs. Baseline in Prograf	-1.005	0.469	0.032	(-1.924, -0.085)
Time: 6m vs. Baseline in Advagraf	-2.043	0.638	0.001	(-3.294, -0.792)
